# Child Defecation and Feces Disposal Practices and Determinants among Households after a Combined Household-Level Piped Water and Sanitation Intervention in Rural Odisha, India

**DOI:** 10.4269/ajtmh.18-0840

**Published:** 2019-02-18

**Authors:** Valerie Bauza, Heather Reese, Parimita Routray, Thomas Clasen

**Affiliations:** 1 Department of Environmental Health, Rollins School of Public Health, Emory University, Atlanta, Georgia;; 2 London School of Hygiene and Tropical Medicine, London, United Kingdom

## Abstract

Latrine access alone may be insufficient to encourage households to dispose of young children’s feces safely in a latrine, and little is known about the determinants of improved child feces disposal. We used longitudinal data collected at up to three timepoints for children less than 5 years of age from households in Odisha, India, which received a combined household-level piped water supply and sanitation intervention, but did not specifically promote the safe disposal of child feces. Among the 85% of intervention households who reported access to improved sanitation, we characterized child defecation and feces disposal practices by age, across time, and season, and assessed determinants of improved disposal. Feces from children less than 3 years of age was commonly picked up by caregivers but disposed of unsafely with garbage into open areas (56.3% of households) or in a drain/ditch (6.2%). Although children 3 and 4 years were more likely to use a latrine than younger children, their feces was also more likely to be left in the open if they did not defecate in a latrine. For children less than 5 years of age, most (84.7%) children’s feces that was safely disposed of in a latrine was because of the children defecating in the latrine directly. Significant predictors for disposing of child feces in an improved latrine were the primary female caregiver reporting using a latrine to defecate, the child’s age, and water observed at place for handwashing. These findings suggest that child feces interventions should focus on encouraging children to begin using a toilet at a younger age and changing the common behavior of disposing of young child’s feces into open areas.

## INTRODUCTION

Globally, an estimated 2.3 billion people lack access to a basic (improved and unshared) sanitation facility, with 892 million people estimated to practice open defecation.^1^ In addition, many households with access to sanitation facilities do not dispose of their young children’s feces in the latrine.^2–4^ Poor child feces management (CFM) presents a particular health risk because young children often have the highest incidence of enteric infections^5^ and poorly developed immune systems, so their feces likely contain higher quantities of transmissible pathogens.^6^ Young children also tend to defecate in areas closer to households, where susceptible children could be exposed,^7^ because young children spend large amounts of time on the ground and commonly engage in exploratory behaviors that include putting fingers, fomites, and soil in their mouths.^8–12^ Consistent with these concerns, unhygienic child feces disposal has been found to be associated with diarrhea,^13^ soil-transmitted helminth infection,^14^ stunting,^3^ and environment enteric dysfunction.^15^

The challenges related to ending open defecation and improving child feces disposal practices are particularly relevant in India, where more than half of the people in the world who practice open defecation reside.^1^ To address these concerns, the Government of India has implemented several national sanitation initiatives, including a series of national sanitation campaigns. However, despite efforts to improve sanitation access, there is evidence of low latrine use.^16–18^ There is also evidence that children’s feces are not commonly disposed of in a latrine, with previous studies in Odisha, India, finding that only about a quarter of households with latrines safely disposed of children’s feces into the latrine in rural study areas^19^ and in urban slum study areas.^4^

To safely manage child feces, all potential points of exposure to pathogens from child feces must be eliminated, including exposure at the sites of defecation, anal cleansing, and feces disposal, as well as the proper cleaning of tools used to assist with defecation or feces disposal, and proper handwashing with soap after feces handling.^4^ However, although recent guidelines on sanitation and health released by the World Health Organization (WHO) note the importance of child defecation, feces disposal, and wash water disposal locations,^20^ current international monitoring focuses solely on the disposal site of child feces. Specifically, the WHO/United Nations International Children’s Emergency Fund (UNICEF) Joint Monitoring Program (JMP) on Water, Sanitation, and Hygiene (WASH) defines “safe” child feces disposal as a child using a toilet facility or the child’s feces being put into a toilet/latrine (regardless of the type of toilet/latrine) or buried. However, a 2015 expert consultation recommended against considering burial as a method of safe disposal because of several reasons, including unknowns about burial practices used and the potential for animals to dig up the feces or rainfall to spread it.^21^ As such, the Water and Sanitation Program (World Bank Group) and UNICEF have alternative classifications, defining “safe” child feces disposal as a child using a toilet facility or the child’s feces being put into any toilet/latrine, and “improved” child feces disposal practices as a child using or his/her feces being put into an improved toilet/latrine^22^; an improved toilet/latrine is defined following JMP guidelines as a flush/pour-flush toilet to a piped sewer system, septic tank, or pit latrine, and a ventilated improved pit latrine, a pit latrine with a slab, or a composting toilet not shared with other households.^23^ In addition, “safely managed” sanitation is also a recently introduced JMP definition that is defined as use of an improved facility with excreta safely disposed of in situ or treated off-site and would be relevant for tracking safe disposal of child feces.^1^

As evident from these definitions, access to a latrine is a necessary condition of safe disposal and CFM. However, increasing latrine access in India does not necessarily translate into latrine use, and latrine use by adults often remains much lower than latrine coverage, with child latrine use and safe disposal of child feces remaining even lower. For example, an evaluation of the Government of India’s Total Sanitation Campaign (TSC) found that latrine coverage increased from 12% in the control group to 63% in the intervention group, but functional latrines with signs of current use only increased from 9% to 36%, and safe disposal of child feces only increased from 3% to 10%.^2,17^ Other barriers to safe child feces disposal may include perceptions that young children’s feces are not harmful,^24,25^ the time, energy, and resources required for safe disposal,^25^ and the practice among individuals in certain castes in India to perform elaborate body cleansing rituals after entering a latrine to dispose of children’s feces.^24^ Lack of water access, especially at the household level, may also be a factor, as water is important for cleaning the latrine slab and flushing of pour-flush toilets, as well as anal cleansing and other cleansing rituals following latrine use in India.^24^ In addition, in some cases, parents may actually discourage children from using a latrine with a squatting slab because they believe that children will dirty the latrine, creating a need for additional cleaning.^26^ Disposal in on-site latrines may also be discouraged because of concerns about pit filling without clear or acceptable options for pit emptying.

The Movement and Action Network for Transformation of Rural Areas (MANTRA) program implemented at the village level by an Indian nongovernmental organization named Gram Vikas attempted to overcome some of the previous shortfalls of sanitation initiatives. The program provided household-level piped water and full community participation in the sanitation initiative by requiring every household in the community to construct a pour-flush latrine and bathing room before turning on the water distribution system to supply piped water. Although the intervention addressed two potential drivers of CFM—latrine and water access—it did not specifically promote safe disposal of child feces. A matched cohort evaluation of this intervention in Odisha several years following the completion of the intervention found higher coverage levels of household piped water and improved toilets, as well as higher toilet use by adults and use for child feces disposal in intervention than control villages.^27^ There was also lower levels of soil-transmitted helminth infection and improved height-for-age *z*-score in intervention villages than control villages, although there was no difference in diarrhea or fecal contamination measured by *Escherichia coli* or *Shigella* spp. in household drinking water or on children’s hands.^27,28^ Although the use of latrines for child feces disposal in intervention villages was higher than that in controls, the levels were still modest, and only approximately one-third of households reported to dispose of their youngest child’s feces in the latrine compared with reported use by approximately three-fourths of older children and adults.^27^ The low levels of child feces disposal practices in latrines in intervention villages could be one reason for the lack of a measured effect of the intervention on diarrhea or measured fecal contamination.

In this study, we used longitudinal data from the matched cohort evaluation to characterize child defecation and feces disposal practices by age, evaluate potential seasonality and consistency of these practices over multiple measurements, and identify determinants of improved child feces disposal. Our primary focus was on analyzing these factors among intervention households with access to improved sanitation, so that infrastructure barriers were not a major factor in poor CFM practices. Our aim is to improve this aspect of CFM programming among households. A better understanding of these factors could improve the design of hardware or educational interventions to target factors that are more likely to improve CFM, as these factors may be different from determinants of improving latrine use by adult household members. Understanding these factors in a community with high levels of coverage of piped water and toilets is critical as poor access to water or toilets would be a barrier to safe CFM and poor CFM could prevent these interventions from fully achieving potential health benefits.

## MATERIALS AND METHODS

### Study site and design.

This sub-study was nested within a matched cohort study evaluating the effectiveness of a household-level piped water and sanitation intervention implemented with complete community coverage in rural Odisha, India. Forty-five control villages were matched with 45 randomly selected intervention villages in the Ganjam and Gajapati districts of Odisha using a matching process that balanced control and intervention arms described in detail in Reese et al.^29^ Within each village, up to 40 households with children less than 5 years of age were randomly selected to be enrolled in the study. The intervention takes an average of 3 years to fully implement and was started in villages between 2003 and 2006. The outcome data were collected for this study to evaluate the intervention between June 2015 and October 2016, enabling an assessment of long-term uptake and effects after the intervention. Because this evaluation was conducted several years after the intervention implementation was complete, the intervention villages did not have complete sanitation coverage at the time of our assessment. The matched cohort study included 1,123 households in intervention villages and 1,275 households in control villages.

As described by Reese et al.,^29^ the MANTRA intervention implemented by Gram Vikas included components of WASH, including 1) household piped water, 2) household pour-flush toilet, and 3) a bathing room attached to the toilet. The piped water was connected to the toilet, bathing room, and kitchen, and the toilet was equipped with dual soak-away pits. For a village to be eligible to participate in the intervention, every household in the village had to agree to construct their own toilet and bathing room (with government incentives covering some or all of the cost for households below the poverty line). Gram Vikas then worked with each village to design and build a piped water system and did not turn on the water distribution system until all households in the village had completed toilet construction. As part of the program, the village creates a corpus fund with contributions from each household, and the village is then responsible for the costs associated with operation and maintenance. Significantly, the intervention did not directly address the safe disposal of child feces or other aspects of safe CFM.

### Household surveys.

Enumerators visited each household up to four times throughout the evaluation, with each study round approximately 4 months apart. Household surveys were primarily administered to the primary caregiver, and data were recorded on mobile phones using the Open Data Kit (available from https://opendatakit.org/). The surveys collected information related to household demographics as well as WASH infrastructure and behaviors. Enumerators also conducted spot-check observations to record information about WASH infrastructure, as well as observe the presence of any feces on the ground in the household compound. Child feces management questions were asked about practices related to the youngest child in each household during survey rounds 1, 3, and 4, including a question related to child feces disposal based on JMP recommendations.^30^ Caregivers were asked “The last time your youngest child under five defecated, where did they defecate?” and “The last time your youngest child under five defecated, what was done to dispose of the stools?” In data collection round 3, households who responded that they disposed of their youngest child’s feces with garbage/solid waste were asked “How do you dispose of your solid waste/garbage?” In addition, although caregivers selected “on a cloth or in the open” as one potential child defecation location during the survey, we have reported this as “on ground or floor” throughout the results for clarity; however, it should be noted that this response may include children who defecated on the ground or floor directly or on a cloth or paper that was laid down. In total, child defecation and feces disposal information was collected for 1,249 control and 1,101 intervention households, which is about 98% of enrolled households. Across rounds, this included 2,501 responses from intervention households (2,124 with and 377 without access to improved sanitation) and 2,848 from control households (605 with and 2,243 without access to improved sanitation) with children less than 5 years of age at the time of data collection.

### Ethical approval.

Informed consent was obtained from the male and/or female head of the household before enrollment. The study was approved by the Ethics Committee of the London School of Hygiene and Tropical Medicine and the Institute Ethics Committee of the Kalinga Institute of Medical Sciences of Kalinga Institute of Industrial Technology (KIIT University) in Bhubaneswar, India. The use of anonymized data for analysis was also approved by the Institutional Review Board of Emory University under a data transfer agreement.

### Statistical analysis.

All analyses were performed in Stata 15.1 (StataCorp LLC, College Station, TX). Except where otherwise noted, the analyses related to CFM were performed using only households in the intervention villages with access to improved sanitation, because access to improved sanitation is necessary to practice improved disposal and few control households had access to latrines. Although children who were 5 years and older did not age out of the matched cohort evaluation if they were aged less than 5 years during enrollment in the first round, we only included the information collected for children who remained less than 5 years of age at the time of data collection for each round in our analysis. We used the Water and Sanitation Program (World Bank Group) and UNICEF definition of improved feces disposal defined in the Introduction section for our analyses.

To explore the effect of potential seasonal variation of child defecation or feces disposal practices between wet season and dry season, we used daily rainfall data for the included villages. Specifically, we used daily gridded satellite precipitation product data with a 0.25° resolution from the Tropical Rainfall Measurement Mission project. Based on these data, wet season was assigned as June 1, 2015 through October 7, 2015 and June 8, 2016 through October 10, 2016. We selected these dates for wet season by taking the summation of daily rainfall for each household in the study area, graphically verifying the strong seasonal trend of rainfall using the 7-day average of daily rainfall, and then assigning wet season to the study period when this 7-day average was frequently (91% of included weeks) above the median and mean rainfall values and dry season to the study period when this 7-day average was frequently (87% of included weeks) below the median and mean values. Chi-squared tests were used to analyze differences in child defecation or feces disposal practices across seasons. The consistency of child defecation and feces disposal practices across data collection rounds was also evaluated using unpaired two-sample *t*-tests.

Logistic regression was used to assess potential determinants of improved child feces disposal among intervention households with improved sanitation and a child less than 5 years of age at the time of the household visit. The following variables were predefined as potential determinants of improved child feces disposal in our model: defecation location for female adults in household, youngest child’s age in years, child’s gender, household wealth quintile, primary female caregiver’s education level, head of household’s education level, number of children less than 5 years of age in the household, whether there was another adult female (older than 18 years) or older children (aged 5–17 years) living in the household who could potentially help with younger children, caregiver’s source of health and nutrition information (with separate variables for sources of family, community health worker, and doctor), head of household’s caste/tribe (categorized as scheduled caste, scheduled tribe, other backward caste, or other caste), whether the female caregiver is one of the people who decides if she can go to place of defecation (as a measure of women’s empowerment), whether the female caregiver is one of the people who decides if she can seek health services (as a measure of women’s empowerment), if animal feces other than pig/dog/monkey/human was observed in the compound (as a measure of compound cleanliness), if the water source is located in their dwelling or on their plot/yard, if the water source was unreliable in the past 24 hours, if the water source was unreliable in the past 2 weeks, and if water was observed at the place for handwashing. Religion was also considered as a potential determinant but was excluded from analysis as more than 96% of intervention households were Hindu. The data for the variables of whether animal feces other than pig/dog/monkey/human was observed in the compound and whether the water source was unreliable in the past 24 hours were only collected during rounds 3 and 4. To enable data from round 1 to also be used in the regression models, a value of “yes” for feces observed was assigned to the variable for round 1 if it was observed in either round 3 or 4 and “no” was assigned to round 1 if it was not observed in both rounds 3 and 4. The same approach was followed for assigning values for unreliable water in the past 24 hours for round 1. All variables were first assessed in a bivariate logistic regression model, and variables with a *P*-value less than 0.2 were included in the multivariable logistic regression model. Robust standard errors in the models were adjusted for clustering at the village level, which is the highest level of clustering present in the data and follows the recommendations of Bottomley et al.^31^ A sensitivity analysis was also performed, adjusting for the household-level clustering instead, which confirmed that adjusting for village-level clustering was a more conservative approach.

## RESULTS

### Household characteristics.

Among intervention households with CFM data collected, 85.0% of households had access to improved sanitation (*N* = 932), 0.1% had access to an unimproved latrine (*N* = 1), and 15.0% reported practicing open defecation (*N* = 164). Levels of open defecation were much higher in control households with CFM data collected, where 78.8% reported practicing open defecation (*N* = 978), 20.9% of households had access to improved sanitation (*N* = 259), and 0.3% of households had access to an unimproved latrine (*N* = 4). Among intervention households with improved sanitation access (the primary households included for our analyses), 81.5% of households had piped water in their own dwelling or on their own yard/plot, 86.6% of households were observed to have water available at their place for handwashing, 87.7% of households reported that male adults (aged 18–59 years) defecated in a latrine, and 93.5% of households reported that female adults (aged 18–59 years) defecated in a latrine, pooling data across data collection rounds. Additional descriptive characteristics of intervention households with access to improved sanitation and CFM data are provided in Supplemental Table 1.

### Child defecation and feces disposal locations by age.

Child defecation and feces disposal locations varied across ages (Table 1; Supplemental Tables 2–5). The most common defecation locations among intervention households with improved sanitation were on the ground or floor (practiced by 55.7% of households with children < 5 years of age), in a toilet/latrine (34.8%), or on clothes (4.5%). Defecating on the ground or floor was the most common defecation location for children less than 3 years of age (practiced by 71.0% of households with children < 3 years of age), and defecating in a toilet or latrine was the most common defecation location for children aged 3 or 4 years (practiced by 67.9% of households with children aged 3 or 4 years). The most common feces disposal locations were to throw feces with garbage (42.2% of households with children < 5 years of age) and to put/rinse feces in the toilet/latrine (40.7%) (Table 1). Other common disposal practices were to leave it in the open (7.0%) or put/rinse feces in a drain/ditch (4.2%). Garbage was the most common disposal location for households with children less than 3 years of age (practiced by 56.3% of households with children < 3 years of age), and it should be noted that households reported the most common garbage disposal location was to dump garbage in an open area (reported by 83% of households; Supplemental Table 6). Feces disposal into a toilet or latrine was the most common disposal location for children aged 3 and 4 years (practiced by 69.2% of households with children aged 3 or 4 years). However, the likeliness of children’s feces being left in the open for children who did not use the latrine also increased with age (3.5% of children’s feces left in open for children aged < 1 year not using the latrine, 6.7% for children aged 1 year, 10.8% for children aged 2 years, 22.5% for children aged 3 years, and 46.6% for children aged 4 years). In addition, there was some variation in the defecation or disposal locations reported by child’s age across rounds (Supplemental Table 2), but the trends were generally consistent. A breakdown of child defecation and feces disposal information for intervention households without improved sanitation and control households with and without improved sanitation is shown in Supplemental Tables 3–5. In households without improved sanitation, young children’s feces are still commonly picked up when a child is young, but the likelihood of a child’s feces to be left in the open increased with age, with 70.1% of feces from children aged 4 years left in the open compared with just 7.4% of feces from children aged 1 year in intervention households without improved sanitation (Supplemental Table 3).

**Table 1:** Child defecation locations by age

	< 1 year (*N* = 297)	1 year (*N* = 562)	2 years (*N* = 531)	3 years (*N* = 416)	4 years (*N* = 318)	All < 5 years (*N* = 2,124)
Defecation location (% of households)						
Toilet/latrine	3.0	9.4	33.7	57.2	81.8	34.8
Potty	1.0	–	–	–	–	0.1
Diaper/nappy	0.7	–	–	–	–	0.1
In clothes	23.9	2.9	1.3	0.2	0.3	4.5
On ground or floor	69.0	82.9	59.5	36.3	13.8	55.7
Other	–	0.4	0.2	0.2	–	0.2
Do not know	2.4	4.5	5.3	6.0	4.1	4.6
Disposal location (% of households)						
Toilet/latrine	24.0	16.8	35.8	58.9	82.7	40.7
Drain/ditch	19.6	2.7	2.5	0.7	–	4.2
Garbage	44.6	69.2	49.2	23.8	4.7	42.2
Buried	0.3	0.4	–	0.2	–	0.2
Left in open	3.4	6.1	7.2	9.6	8.5	7.0
Other	6.1	0.5	0.4	0.5	–	1.2
Do not know	2.0	4.5	5.1	6.3	4.1	4.6

The results presented are for intervention households with improved sanitation and include data from all relevant rounds of collection.

### Relationship between child defecation and feces disposal practices.

When analyzing feces disposal methods by defecation location for intervention households with improved sanitation, the majority of feces from children defecating on the ground or floor was disposed of with garbage (73.3%), with feces being left in the open being the second most common disposal method for this defecation location (12.0%; Table 2). For children who defecated on clothes, disposal in a toilet or latrine (36.5%), in a ditch/drain (30.2%), and with garbage (19.8%) were all common methods (Table 2). When analyzing defecation location by feces disposal method, the majority (84.7%) of the total children with feces disposed of in a toilet or latrine resulted from children who defecated there (Table 2). On the other hand, most feces that were disposed of in a drain/ditch, with garbage, buried, or left in the open came from children who defecated on the ground or floor (Table 2).

**Table 2:** Breakdown of disposal locations for each defecation location (upper half of table) and defecation locations for each disposal location (lower half of table) for households with children less than 5 years of age

Defecation location:	Toilet/latrine (*N* = 739)	Potty (*N* = 3)	Diaper/nappy (*N* = 2)	Clothes (*N* = 96)	On ground or floor (*N* = 1,181)	Other (*N* = 4)	Do not know (*N* = 97)
Disposal location (% of defecation location)							
Toilet/latrine	98.9	0	50.0	36.5	8.0	0	1.0
Drain/ditch	0	33.3	0	30.2	4.9	0	1.0
Garbage	1.0	0	50.0	19.8	73.3	50.0	0
Buried	0	0	0	0	0.3	0	0
Left in open	0	33.3	0	5.2	12.0	25.0	0
Other	0	33.3	0	8.3	1.3	25.0	0
Do not know	0.1	0	0	0	0.1	0	97.9

Disposal location:	Toilet/latrine (*N* = 863)	Drain/ditch (*N* = 89)	Garbage (*N* = 895)	Buried (*N* = 4)	Left in open (*N* = 149)	Other (*N* = 25)	Do not know (*N* = 97)
Defecation location (% of disposal location)							
Toilet/latrine	84.7	0	0.8	0	0	0	1.0
Potty	0	1.1	0.1	0	0.7	4.0	0
Diaper/nappy	0.1	0	0	0	0	0	0
Clothes	4.1	32.6	2.1	0	3.4	32.0	0
On ground or floor	11.0	65.2	96.8	100	95.3	60.0	1.0
Other	0	0	0.2	0	0.7	4.0	0
Do not know	0.1	1.1	0	0	0	0	97.9

These results are for intervention households with improved sanitation and include data from all relevant rounds of collection.

### Seasonal variation of child defecation and feces disposal practices.

There was no difference observed between the defecation locations of children less than 5 years of age in intervention households with improved sanitation reported for dry season versus wet season (χ^2^ = 5.5, *P* = 0.48; Supplemental Table 7). There was a statistically significant difference between the feces disposal locations reported for children less than 5 years of age for dry season versus wet season (χ^2^ = 14.8, *P* = 0.022), with slightly more children’s feces left in the open during dry season and slightly more children’s feces disposed of with garbage in the wet season; however, this difference was likely not large enough to be meaningful (Supplemental Table 7).

### Longitudinal analysis of child defecation and feces disposal practices.

Among intervention households with access to improved sanitation and children less than 5 years of age, approximately 60.3% of households had CFM data collected at all three data collection rounds. Among these households, only 44.3% of them reported the same location for child defecation at all rounds and 29.9% reported the same location for child feces disposal at all rounds. This proportion of households with consistent child feces disposal practices was significantly less than the proportion of households with consistent child defecation practices (*t* = 5.08, *P* < 0.0001). In addition, the low proportion of households reporting consistent feces disposal practices across rounds was similar, regardless of which households were analyzed, with this proportion ranging between 28% and 30% when analyzing all study households and households grouped by intervention or control status, or access to improved or unimproved sanitation. The trend of a greater proportion of households reporting consistent child defecation practices compared with feces disposal practices was also consistent across these groups, although a greater proportion of households reported consistent child defecation practices among households that did not have access to improved sanitation compared with households that did (69.5% of intervention households without improved sanitation reported consistent child defecation location across the three rounds, with the child consistently defecating on the ground or floor). For intervention households with access to improved sanitation, the most common consistent defecation location was on the ground or floor (72.6%), followed by in the latrine (27.0%), and the most common consistent disposal location was with garbage (50.6%), followed by in the latrine (47.1%; Table 3).

**Table 3:** Longitudinal analysis of defecation and feces disposal locations by consistency of the reported practices across the rounds of data collection

	Consistent	Inconsistent, round no.		
		1	3	4
Defecation location (% of households)				
*N*	252	317		
Toilet/latrine	27.0	24.0	43.2	42.9
Potty	–	–	0.3	0.3
Diaper/nappy	–	0.3	–	0.3
In clothes	0.4	9.2	5.7	5.1
On ground or floor	72.6	62.2	38.2	32.2
Other	–	0.3	1.3	5.1
Do not know	–	4.1	11.4	14.2
Disposal location (% of households)				
*N*	170	399		
Toilet/latrine	47.1	26.1	35.8	39.6
Drain/ditch	–	5.8	6.5	5.3
Garbage	50.6	54.1	33.6	29.6
Buried	–	0.5	0.3	–
Left in open	2.4	7.5	12.8	10.8
Other	–	2.5	1.8	3.8
Do not know	–	3.5	9.3	11.0

These results are for intervention households with improved sanitation and children less than 5 years of age and include data from all relevant rounds of collection.

### Determinants of improved child feces disposal practices.

In the unadjusted bivariate logistic regression analysis of each variable as a potential determinant of improved child feces disposal, the following variables were significant: female adults in household defecate in toilet (odds ratio [OR] = 6.84, 95% CI = 3.51–13.3), child age (children aged 1–2 years: OR = 0.65, 95% CI = 0.44–0.95; children aged 2–3 years: OR = 1.83, 95% CI = 1.31–2.56; children aged 3–4 years: OR = 5.10, 95% CI = 3.45–7.54; children aged 4 to < 5 years: OR = 19.2, 95% CI = 12.8–28.9; reference: children aged < 1 year), number of children less than 5 years of age in household (OR = 0.57, 95% CI = 0.48–0.67), head of household has received some formal education (OR = 1.27, 95% CI = 1.01–1.60), animal feces (other than pig/dog/monkey/human) observed in compound (OR = 0.62, 95% CI = 0.48–0.81), water located in own dwelling or yard/plot (OR = 1.71, 95% CI = 1.19–2.46), water observed at the place of handwashing (OR = 2.02, 95% CI = 1.49–2.74), and caregiver is one of the people who decides if she can go to the place of defecation (OR = 1.49, 95% CI = 1.14–1.96). The full results of the bivariate logistic regressions are available in Supplemental Table 8.

In the adjusted multivariable model, only the variables for female adults in household defecate in toilet (OR = 5.68, 95% CI = 2.19–14.8), child age (children aged 1–2 years: OR = 0.41, 95% CI = 0.25–0.65; children aged 3–4 years: OR = 3.77, 95% CI = 2.30–6.18; children aged 4 to < 5 years: OR = 22.8, 95% CI = 10.4–49.9; reference: children aged < 1 year), and water observed at the place of handwashing (OR = 1.78, 95% CI = 1.12–2.82) remained significant (Table 4). In addition, the variable for the head of household from an “other backward caste” was significant in the adjusted model (OR = 0.61, 95% CI = 0.38–0.98, reference: caste other than scheduled caste, scheduled tribe, or other backward caste), but the other household caste/tribe variables were not.

**Table 4:** Logistic regression results for adjusted analysis of potential determinants of improved child feces disposal

	Adjusted odds ratio	95% CI	*P*-value
Female adults in household defecate in toilet	5.68	2.19–14.8	< 0.001*
Child age (ref. < 1 year)			
1–2 years	0.41	0.25–0.65	< 0.001*
2–3 years	1.37	0.86–2.17	0.182
3–4 years	3.77	2.30–6.18	< 0.001*
4 to < 5 years	22.8	10.4–49.9	< 0.001*
Number of children < 5 years of age in the household	0.85	0.68–1.06	0.138
Household wealth quintiles (ref. poorest)			
Poorer quintile	1.13	0.77–1.67	0.527
Middle quintile	1.04	0.67–1.63	0.853
Richer quintile	0.92	0.59–1.43	0.702
Richest quintile	0.63	0.39–1.00	0.050
Head of household received any formal education	1.39	0.96–2.01	0.084
Household caste/tribe (ref. other caste)			
Scheduled caste	0.69	0.36–1.33	0.265
Scheduled tribe	1.06	0.44–2.57	0.897
Other backward caste	0.61	0.38–0.98	0.043*
Animal feces (other than pig/dog/monkey) observed in compound	0.75	0.50–1.13	0.171
Water located in own dwelling or yard/plot	1.32	0.77–2.27	0.308
Water source unreliable in the past 2 weeks	0.62	0.35–1.12	0.116
Water observed at place for handwashing	1.78	1.12–2.82	0.014*
Caregiver is one of the people who decides if she can go to the place of defecation	0.99	0.67–1.45	0.953
Constant	0.080	0.016–0.40	0.002*

The data analyzed included data from all rounds of data collection from intervention households with improved sanitation and children less than 5 years of age at the time of data collection.

* Significant, *P* < 0.05.

We also compared the proportion of women who defecate in a latrine who also dispose of their youngest child’s feces in a latrine among intervention with control households and found no difference in this proportion (40.9% of women who defecate in a latrine in the control households also disposed of their youngest child’s feces in the latrine compared with 42.7% in intervention households, χ^2^ = 0.57, *P* = 0.45).

## DISCUSSION

This work found evidence that children’s defecation and feces disposal practices change with age, that most children’s feces that was disposed of in a toilet originated from children defecating there, that feces disposal practices are inconsistent over time, and that there were minimal differences in child defecation or feces disposal practices across season in this rural India setting. These findings have implications for the design of future interventions to encourage child feces disposal and suggest that interventions which encourage children to use the latrine directly may be potentially beneficial interventions that are often overlooked when discussing CFM. This study also evaluated potential determinants of improved child feces disposal among households in villages following an intervention for household piped water and pour-flush toilets, and no obvious determinants were identified that can be targeted in future interventions. Consistent with several other studies,^2,3,19,25,32,33^ we also found that many households with access to a toilet still do not dispose of their children’s feces in the toilet, providing additional support for the necessity of separate interventions which may include targeted behavior change and CFM hardware to improve CFM that go beyond toilet and water access.

Our findings have important implications for CFM interventions as they indicate that typical interventions may not target behaviors related to CFM most likely to contaminate the environment. Typical CFM interventions involve the distribution and/or promotion of scoops to pick up feces off the ground, diapers/nappies for infants to defecate into, and/or potties for toddlers to defecate into.^25,34,35^ These types of interventions are typically targeted at children less than 3 years of age. Specifically, interventions aimed at children less than 3 years of age may fail to target the children most likely to have their feces left in the open after defecation, which was found to be the case for children aged three and four in our study location, particularly when these children did not use the latrine directly. In addition, interventions targeting the defecation location or process of picking up feces for children less than 3 years of age may not alter the disposal location, which was found to be with garbage for most of the children less than 3 years of age in our study. On the other hand, defecating in a latrine was a strong predictor of improved disposal into a latrine, with defecating in a latrine being the defecation location for 85% of all children’s feces safely disposed of. This is consistent with a past study in rural Odisha that found less than 1% of households receiving the Government of India’s TSC intervention as part of a randomized controlled trial reported disposing of children’s feces in a latrine if the child did not defecate there themselves.^2^ This finding suggests that getting children to begin using a toilet at a younger age could improve child feces disposal practices and interventions are needed to help with these practices. This may require improvements in latrine design and lighting, as well as changes in behavior and norms. Seats fitted over the pan of pour-flush or flush latrines may also help encourage children to begin using a latrine at a younger age.^25^ However, more research is needed to identify effective child-friendly latrine designs.

Our results also highlight that feces of children less than 3 years of age were usually picked up by caregivers. However, the main concern with feces disposal practices for children less than 3 years of age in this setting was that the feces were commonly disposed of with garbage, which was reported by the vast majority of respondents (83%) to be dumped into an open area with a potential to contaminate the environment. Although solid waste infrastructure and practices related to garbage disposal vary in different settings, the JMP considers disposal of human feces with solid waste/garbage to be classified as open defecation for Sustainable Development Goal monitoring.^1^ The introduction of the common feces management tools of scoops, diapers/nappies, and potties aim to change a child’s defecation location, but would not target changing this unsafe disposal behavior of disposing of feces with garbage in an open area. Interventions that are instead targeted at changing this disposal behavior may be more effective at targeting the specific unsafe behavior in the CFM chain.

There were no meaningful differences observed between the reported child defecation or feces disposal locations during dry season or wet season. Differences may be expected in some locations if raining floods a household’s latrine or prevents households from traveling to their typical defecation location. Rain during the wet season may also be more likely to spread contamination from the feces of children who defecate in the open. The lack of a difference between practices across seasons indicates that CFM interventions in this region may not need to change during the wet season. However, it is also possible that rain could have more of an effect on CFM practices if it is raining on the particular day and at the particular time that a child defecates than if it is during wet season or dry season.

Longitudinal analysis of data collected over multiple study rounds indicated that feces disposal practices were often inconsistent across time and were more likely to be inconsistent than defecation location. In some cases, practices may be changing over time as a child gets older or when a new child is born into the households. For example, in households with inconsistent practices, many reported greater use of latrines by children in later rounds, possibly because of children getting older. It is also possible that respondents could have been anticipating the “desirable” answer in follow-up visits, which could have also made them more likely to report hygienic behaviors in later rounds. Some practices for the youngest child in the household could have also changed among households because of a new child being born, as new children were born into approximately 11% of these households between rounds 1 and 4. However, some of this reported inconsistency may be due to varied practices, with caregivers sometimes disposing of feces in one location and disposing of it in another location at other times. To better characterize feces disposal locations in future studies, we recommend the addition of a question to surveys that ask caregivers if there are any other locations that they commonly use to dispose of their child’s feces to determine if a household engages in other disposal practices that could potentially contaminate the environment.

Analysis of determinants found that the defecation practice for the primary female caregiver, the age of the child, and whether the handwashing facility had water were significant predictors of improved child feces disposal. Caste or tribe of the head of households could potentially play a role as “other backward caste” was found to be a significant predictor, but not other caste or tribe variables. Wealth was not found to be a significant predictor of improved disposal, suggesting that wealth is not a main factor in determining feces disposal practices in a setting such as this, where households in intervention villages had access to household piped water and participated in constructing household latrines, regardless of wealth. This analysis also revealed that there are not any obvious determinants to focus on to improve child feces disposal practices in this location, where access to piped water and sanitation infrastructure were not barriers. The defecation location for the primary female caregiver was a strong predictor of improved feces disposal, so interventions that improve toilet use by caregivers may also improve child feces disposal practices, but this alone is unlikely to have a meaningful impact on disposal practices, as only about 40% of caregivers who reported defecating in a latrine also disposed of their children’s feces there. Similarly, having water at a handwashing station was also a predictor of improved child feces disposal practices, likely because caregivers who could wash their hands after handling their children’s feces were more likely to dispose of it in a toilet. However, this factor is also unlikely to have a high impact on feces disposal practices on its own, as only around 40% of households that had water at their handwashing station practiced safe disposal. The age of a child was also a strong predictor, which is consistent with children being more likely to use a toilet themselves as they get older, which would lead to safe disposal practices. Hardware or behavioral interventions that encourage greater use of toilets by young children and encourage children to use a toilet consistently at a younger age may have a meaningful impact on improving child feces disposal practices. Past studies have also found child feces disposal to be associated with the child’s age, toilet/latrine access, household adults using latrine for defecation, household wealth, caregiver’s education, head of household’s education, caregiver’s age, and water source,^2,25,32,33^ which is generally consistent with our results.

In addition, when comparing intervention with control households, we found no difference in the proportion of women who defecate in a toilet who also dispose of their youngest child’s feces in a toilet. As female caregivers are often responsible for CFM, this finding suggests that there were likely behavioral or other determinants of safe child feces disposal that were already present in women before the intervention was delivered, and these determinants motivated safe child feces disposal practices once toilet access was achieved. However, it is unlikely that the intervention itself improved these behaviors beyond creating toilet and water access.

### Limitations.

This study had several limitations. First, we only collected child feces defecation and disposal data for the youngest child less than 5 years of age, and there may be different practices for different children in household. In addition, children defecating either “on a cloth or in the open” was recorded as the same response that we interpreted as on the ground or floor. We did not collect separate information from children defecating on the ground directly about caregivers laying down paper or cloth before a child defecates, so we cannot comment on the prevalence of these separate defecation practices or if using a cloth was associated with safer disposal of child feces than defecating on the ground directly. However, it is likely that the number of children defecating on a cloth was relatively low, as previous studies reported no children defecating on cloth and less than 5% of children less than 5 years of age defecating on paper in rural Odisha^19^ and less than 5% of child less than 5 years of age defecating on cloth on the ground inside or outside in urban slums of Odisha.^4^ An additional limitation of our study is that CFM practices were self-reported, which could have led to bias and potential overreporting of hygienic behaviors. Past studies have observed that caregivers are more likely to report the perceived desirable behavior of disposing of child feces in a latrine when asked during questionnaires compared with the same caregivers observed to actually put their child’s feces in a latrine during structured observation.^36,37^

Our study focused on child defecation and feces disposal locations, which are two important CFM behaviors for determining the potential of contamination and exposure from CFM practices, but there are also other practices along the chain of events related to CFM that could contaminate the environment. This includes practices related to how the ground is cleaned after defecation, whether caregiver’s hands are washed after contact with children’s feces, how and where anal cleansing of the child is performed, how nappies are cleaned or diapers disposed of, and how scoops or potties are washed and where wash water is disposed of if these tools are used. In addition, whereas we asked how garbage was disposed of, how garbage is stored and handled could also be important factors in determining potential exposure, especially if garbage is stored in a manner that children, flies, or animals could come in contact with it. Our study allows the characterization of potential risks and practices for two behaviors (defecation and feces disposal) assumed to be most likely to cause exposure or contamination along the CFM chain, but future work should also consider other activities along this chain of events to make evidence-based recommendations of best practices and potential interventions.

## CONCLUSION

Disposal of child feces into a latrine was uncommon, even among households with access to an improved pour-flush latrine that was used by adults in the household. Two important findings were that the feces from children less than 3 years of age was commonly picked up by caregivers but disposed of unsafely with garbage into open areas and that most children’s feces that was safely disposed of in a toilet was because of children defecating in the toilet directly. These findings suggest there may be a need to rethink traditional child feces interventions of scoops, diapers/nappies, and potties for children less than 3 years of age, as these may fail to alter the current practices of disposing of feces with garbage, a common practice for children less than 3 years of age in this setting in rural India. These findings also suggest that interventions which are effective at encouraging children to begin using a toilet at a younger age could improve child feces disposal practices. More research is needed to evaluate the most effective interventions for promoting safe CFM practices in ways that will achieve sustained uptake, with a focus on practices that encourage use of latrines earlier by children and shift from disposal of feces in garbage to disposal in a toilet.

## Supplementary Files

Supplemental tables
