# Supplementary material for: Child Defecation and Feces Disposal Practices and Determinants among Households after a Combined Household-Level Piped Water and Sanitation Intervention in Rural Odisha, India
Source: Am J Trop Med Hyg. 2019 Feb 18;100(4):1013–21. doi: 10.4269/ajtmh.18-0840 (PMC6447099; doi:10.4269/ajtmh.18-0840)
Supplement: Supplementary file 1 [file tpmd180840.SD1.pdf]

SUPPLEMENTAL INFORMATION

Child defecation and feces disposal practices and determinants among households after a combined household-level piped water and sanitation intervention in rural Odisha, India

Valerie Bauza,[1,*] Heather Reese, [1] Parimita Routray, [2] Thomas Clasen[1]

[1] Department of Environmental Health, Rollins School of Public Health, Emory University, Atlanta, Georgia, United States of America
[2] London School of Hygiene and Tropical Medicine, London, United Kingdom

*Corresponding author email: valerie.bauza@emory.edu

Tables

Table S1: Descriptive characteristics of intervention households with improved sanitation and child feces management (CFM) data
Table S2: Child defecation and disposal locations by age across rounds for intervention households with improved sanitation.
Table S3: Child defecation and disposal locations by age for intervention households without improved sanitation.
Table S4: Child defecation and disposal locations by age for control households with improved sanitation.
Table S5: Child defecation and disposal locations by age for control households without improved sanitation.
Table S6: Solid waste disposal location for garbage.
Table S7: Child defecation and feces disposal locations by season for children under five in intervention households with improved sanitation.
Table S8: Results of unadjusted bivariate logistic regressions analyzing for potential determinants of improved child feces disposal.

Table S1. Descriptive characteristics of intervention households with improved sanitation and child feces management (CFM) data

Total observations across rounds, N	2,124
Female adults in household defecate in toilet, N (%)	1,980 (93.6%)
Child age (youngest child)	
< 1 year, N (%)	297 (14.0%)
1-2 years, N (%)	562 (26.5%)
2-3 years, N (%)	531 (25.0%)
3-4 years, N (%)	416 (19.6%)
4-<5 years, N (%)	318 (15.0%)
Child sex is female, N (%)	974 (46.3%)
Number of children under 5 in household, mean (sd)	2.6 (0.8)
At least one older child or a 2nd female adult is present in the household, N (%)	1,674 (78.8%)
Household wealth quintiles	
Poorest quintile, N (%)	327 (15.4%)
Poorer quintile, N (%)	357 (16.8%)
Middle quintile, N (%)	403 (19.0%)
Richer quintile, N (%)	448 (21.1%)
Richest quintile, N (%)	589 (27.7%)
Female caregiver received any formal education, N (%)	1,357 (73.6%)
Head of household received any formal education, N (%)	1,014 (59.6%)
Household caste/tribe	
Scheduled caste, N (%)	194 (10.2%)
Scheduled tribe, N (%)	238 (12.5%)
Other backward caste, N (%)	810 (42.6%)
Other caste, N (%)	660 (34.7%)
Animal feces (other than pig/dog/monkey) observed in compound (excluding assumed values for round 1), N (%)	310 (23.1%)
Water located in own dwelling or yard/plot, N (%)	1,722 (81.4%)
Water source unreliable in the past 24 hours (excluding assumed values for round 1), N (%)	324 (23.9%)
Water source unreliable in the past 2 weeks, N (%)	265 (12.5%)
Water observed at place for handwashing, N (%)	1,816 (86.7%)
Women gets health info from:	
Family, N (%)	480 (26.0%)
Community health worker, N (%)	1,144 (61.8%)
Doctor, N (%)	1,053 (56.9%)
Caregiver is one of the people who decides if:	
She can go to place of defecation, N (%)	908 (56.0%)
She can seek health services, N (%)	884 (54.5%)

Table S2. Child defecation and disposal locations by age across rounds for intervention households with improved sanitation.

	<1 yr			1 yr			2 yr			3 yr			4 yr		
Round #	1	3	4	1	3	4	1	3	4	1	3	4	1	3	4
N	158	77	62	206	198	158	173	180	178	138	140	138	95	107	116
**Defecation location (% of households)**															
Toilet/latrine	0.6%	3.9%	8.1%	7.3%	12.1%	8.9%	34.1%	37.2%	29.8%	58.0%	61.4%	52.2%	77.9%	79.4%	87.1%
Potty	0.6%	1.3%	1.6%	-	-	-	-	-	-	-	-	-	-	-	-
Diaper/nappy	0.6%	-	1.6%	-	-	-	-	-	-	-	-	-	-	-	-
In clothes	23.4%	23.4%	25.8%	3.9%	2.0%	2.5%	1.2%	1.1%	1.7%	-	0.7%	-	-	-	0.9%
On ground or floor	72.2%	71.4%	58.1%	85.0%	80.8%	82.9%	63.6%	52.2%	62.9%	41.3%	30.7%	37.0%	17.9%	15.9%	8.6%
Other	-	-	-	0.5%	-	0.6%	-	-	0.6%	0.7%	-	-	-	-	-
Don’t know	2.5%	-	4.8%	3.4%	5.1%	5.1%	1.2%	9.4%	5.1%	-	7.1%	10.9%	4.2%	4.7%	3.5%
**Feces disposal location (% of households)**															
Toilet/latrine	24.2%	23.4%	24.2%	18.5%	16.2%	15.2%	34.1%	37.2%	36.0%	57.3%	61.4%	58.0%	76.8%	81.3%	88.8%
Drain/ditch	13.4%	27.3%	25.8%	2.0%	1.5%	5.1%	1.7%	3.3%	2.3%	-	0.7%	1.5%	-	-	-
Garbage	47.8%	39.0%	43.6%	71.7%	68.7%	66.5%	54.3%	43.9%	49.4%	31.2%	20.0%	20.3%	7.4%	1.9%	5.2%
Buried	-	1.3%	-	1.0%	-	-	-	-	-	0.7%	-	-	-	-	-
Left in open	1.9%	6.5%	3.2%	2.4%	8.1%	8.2%	8.1%	6.1%	7.3%	10.1%	10.0%	8.7%	11.6%	12.2%	2.6%
Other	10.2%	2.6%	-	1.0%	0.5%	-	0.6%	-	0.6%	0.7%	0.7%	-	-	-	-
Don’t know	2.6%	-	3.2%	3.4%	5.1%	5.1%	1.2%	9.4%	4.5%	-	7.1%	11.6%	4.2%	4.7%	3.5%

	All children <5 yr		
Round #	1	3	4
N	770	702	652
**Defecation location**			
Toilet/latrine	29.7%	37.8%	37.6%
Potty	0.1%	0.1%	0.2%
Diaper/nappy	0.1%	-	0.2%
In clothes	6.1%	3.6%	3.7%
On ground or floor	61.4%	52.6%	52.2%
Other	0.3%	-	0.3%
Don’t know	2.2%	6.0%	6.0%
**Feces disposal location**			
Toilet/latrine	37.4%	41.3%	43.9%
Drain/ditch	3.7%	4.4%	4.6%
Garbage	47.7%	39.2%	39.0%
Buried	0.4%	0.1%	-
Left in open	6.1%	8.4%	6.6%
Other	2.6%	0.6%	0.2%
Don’t know	2.2%	6.0%	5.8%

Table S3. Child defecation and disposal locations by age for intervention households without improved sanitation.

	**<1 yr** (N=61)	**1 yr** (N=95)	**2 yr** (N=76)	**3 yr** (N=68)	**4 yr** (N=77)	**All <5 yr** (N=377)
**Defecation location (% of households)**						
Toilet/latrine	-	1.1	1.3	1.5	1.3	1.1
Potty	-	-	-	-	-	-
Diaper/nappy	-	-	-	-	-	-
In clothes	19.7	1.1	-	-	-	3.5
On ground or floor	80.3	95.8	97.4	98.5	94.8	93.9
Other	-	-	-	-	-	-
Don't know	-	2.1	1.3	-	3.9	1.6
**Feces disposal location (% of households)**						
Toilet/latrine	3.3	1.1	1.3	1.5	1.3	1.6
Drain/ditch	34.4	6.3	2.6	1.5	-	8.0
Garbage	49.2	83.2	73.7	61.8	24.7	60.0
Buried	-	-	1.3	-	-	0.3
Left in open	3.3	7.4	19.7	35.3	70.1	27.1
Other	9.8	-	-	-		1.6
Don't know	-	2.1	1.3	-	3.9	1.6

Table S4. Child defecation and disposal locations by age for control households with improved sanitation.

	**<1 yr** (N=74)	**1 yr** (N=177)	**2 yr** (N=139)	**3 yr** (N=127)	**4 yr** (N=88)	**All <5 yr** (N=605)
**Defecation location (% of households)**						
Toilet/latrine	4.1	12.4	30.2	51.2	77.3	33.1
Potty	-	0.6	1.4	0.8	-	0.7
Diaper/nappy	-	-	-	-	-	-
In clothes	25.7	3.4	0.7	0.8	2.3	4.8
On ground or floor	63.5	78.0	61.9	37.8	19.3	55.5
Other	-	0.6	0.7	0.8	-	0.5
Don't know	6.8	5.1	5.0	8.7	1.1	5.5
**Feces disposal location (% of households)**						
Toilet/latrine	12.2	17.0	34.5	51.2	78.4	36.5
Drain/ditch	21.6	4.5	0.7	-	1.1	4.3
Garbage	48.7	65.5	50.4	29.1	10.2	44.3
Buried	-	0.6	-	1.6	-	0.5
Left in open	4.1	5.7	7.9	8.7	9.1	7.1
Other	6.8	1.7	1.4	0.8	-	1.8
Don't know	6.8	5.1	5.0	8.7	1.1	5.5

Table S5. Child defecation and disposal locations by age for control households without improved sanitation.

	**<1 yr** (N=391)	**1 yr** (N=549)	**2 yr** (N=541)	**3 yr** (N=424)	**4 yr** (N=338)	**All <5 yr** (N=2,243)
**Defecation location (% of households)**						
Toilet/latrine	-	-	0.4	0.2	0.6	0.2
Potty	-	-	-	-	-	0.04
Diaper/nappy	-	-	-	-	-	-
In clothes	25.6	1.8	0.9	0.2	0.3	5.2
On ground or floor	72.1	95.8	95.8	95.3	95.9	91.6
Other	1.0	-	0.2	0.7	0.6	0.5
Don't know	1.3	2.4	2.8	3.5	2.7	2.5
**Feces disposal location (% of households)**						
Toilet/latrine	-	-	0.4	-	0.6	0.2
Drain/ditch	34.0	4.2	1.3	0.9	1.2	7.6
Garbage	45.5	78.1	69.0	44.6	29.0	56.5
Buried	-	-	0.4	0.2	-	0.1
Left in open	7.7	13.5	25.7	50.0	66.3	30.3
Other	11.5	1.6	0.6	0.7	0.6	2.8
Don't know	1.3	2.6	2.8	3.5	2.4	2.5

Table S6. Solid waste disposal location for garbage. Results from round 3 of surveys for intervention households with improved sanitation that reported disposal of child feces with garbage.

**Solid waste disposal location**	**N (%)**
Dump to open area	229 (83.3%)
Street containers – open top	1 (0.4%)
Burn	10 (3.6%)
Compost	25 (9.1%)
Other	10 (3.6%)

Table S7. Child defecation and feces disposal locations by season for children under five years in intervention households with improved sanitation.

	Dry season N=836	Wet season N=1,295
**Defecation location (% of households)**		
Toilet/latrine	36.6%	33.7%
Potty	0.1%	0.2%
Diaper/nappy	0.1%	0.1%
Clothes	3.8%	4.9%
On ground or floor	53.8%	56.9%
Other	0.2%	0.2%
Don't know	5.4%	4.1%
**Disposal location (% of households)**		
Toilet/latrine	40.8%	40.6%
Drain/ditch	4.4%	4.0%
Garbage	39.8%	43.8%
Buried	0.1%	0.2%
Left in open	8.9%	5.8%
Other	0.6%	1.6%
Don't know	5.4%	4.0%

Table S8. Results of unadjusted bivariate logistic regressions analyzing for potential determinants of improved child feces disposal. The data analyzed included data from all rounds of data collection from intervention households with improved sanitation and children under 5 at the time of data collection.

Variable	OR	95% CI	p-value
Female adults in household defecate in toilet	6.84	3.51 – 13.3	<0.001*
Child age (ref. <1 yr)			
1-2 yr	0.65	0.44 – 0.95	0.026
2-3 yr	1.83	1.31 – 2.56	<0.001*
3-4 yr	5.10	3.45 – 7.54	<0.001*
4-<5 yr	19.2	12.8 – 28.9	<0.001*
Child sex is female	1.10	0.88 – 1.37	0.409
Number of children under 5 in household	0.57	0.48 – 0.67	<0.001*
At least one older child or a 2nd female adult is present in the household	1.20	0.90 – 1.60	0.211
Household wealth quintiles (ref. poorest)			
Poorer quintile	1.34	0.92 – 1.95	0.133
Middle quintile	1.39	0.97 – 1.98	0.076
Richer quintile	1.34	0.95 – 1.88	0.099
Richest quintile	1.19	0.82 – 1.70	0.358
Female caregiver received any formal education	1.17	0.87 – 1.57	0.295
Head of household received any formal education	1.27	1.01 – 1.60	0.045*
Household caste/tribe (ref. other caste)			
Scheduled caste	0.65	0.39 – 1.09	0.101
Scheduled tribe	0.57	0.29 – 1.10	0.092
Other backward caste	0.76	0.54 – 1.07	0.115
Animal feces (other than pig/dog/monkey) observed in compound	0.62	0.48 – 0.81	<0.001*
Water located in own dwelling or yard/plot	1.71	1.19 – 2.46	0.004*
Water source unreliable in the past 24 hours	0.89	0.72 – 1.11	0.314
Water source unreliable in the past 2 weeks	0.81	0.60 – 1.11	0.185
Water observed at place for handwashing	2.02	1.49 – 2.74	<0.001*
Women gets health info from:			
Family	1.02	0.79 – 1.32	0.875
Community health worker	1.11	0.86 – 1.44	0.419
Doctor	0.94	0.76 – 1.17	0.595
Caregiver is one of the people who decides if:			
She can go to place of defecation	1.49	1.14 – 1.96	0.004*
She can seek health services	1.15	0.83 – 1.60	0.388

* significant, p<0.05
OR = odds ratio, CI = confidence interval
